# Supplementary material for: Epstein-Barr virus protein EBNA-LP engages YY1 through leucine-rich motifs to promote naïve B cell transformation
Source: PLoS Pathog. 2024 Jul 31;20(7):e1011950. doi: 10.1371/journal.ppat.1011950 (PMC11318927; doi:10.1371/journal.ppat.1011950)
Supplement: S1 Text — (DOCX) [file ppat.1011950.s001.docx]

Identification of EBNA-LP Phosphorylation Sites by LC-MS/MS

The sample, elution of FLAG Immunoprecipitation from wild type EBNA-LP expressed in 293T cells, was brought to 4% SDS, reduced with 10 mM dithiolthreitol for 20 min at 55°C, alkylated with 25mM iodoacetamide for 45 min at room temperature and then subjected to S-trap (Protifi) trypsin digestion using manufacturer recommended protocols. Digested peptides were lyophilized to dryness and resuspended in 12 μL of 0.2% formic acid (FA)/2% acetonitrile (ACN). The sample was subjected to chromatographic separation on a Waters MClass UPLC equipped with a 1.8 μm Acquity HSS T3 C18 75 μm × 250 mm column (Waters Corp.) with a 90-min linear gradient of 5 to 30% ACN with 0.1% FA at a flow rate of 400 nanoliters/minute (nL/min) with a column temperature of 55°C. Data collection on the Fusion Lumos mass spectrometer in a data-dependent acquisition (DDA) mode of acquisition with a r=120,000 (@ m/z 200) full MS scan from m/z 375 – 1500 with a target AGC value of 4e5 ions was performed. MS/MS scans with HCD settings of 30% were acquired in the linear ion trap in “rapid” mode with a target AGC value of 1e4 and max fill time of 35 ms. The total cycle time was 2 sec between like full MS scans. A 20s dynamic exclusion was employed to increase depth of coverage. The total analysis cycle time for each sample injection was approximately 2 hours.

Raw LC-MS/MS data files were processed in Mascot Distiller and then submitted to independent Mascot database searches against a *Human* protein database containing both forward and reverse entries of each protein as well as EBNA-LP. Search tolerances were 2 ppm for precursor ions and 0.8 Da for product ions using trypsin specificity with up to two missed cleavages. Carbamidomethylation (+57.0214 Da on C) was set as a fixed modification, whereas oxidation (+15.9949 Da on M), phosphorylation (+79.97 Da on STY), hydroxyprolination (+15.99 Da on P) and acetylation (+42.01 Da on K) were considered as dynamic mass modifications. All searched spectra were imported into Scaffold (v5.3, Proteome Software) and scoring thresholds were set to achieve a peptide false discovery rate of 1% using the PeptideProphet algorithm. PTM localization data were assigned through Scaffold PTM using the AScore algorithm.

**Quantification of Phosphorylation Sites on EBNA-LP Wild Type, -S3A, -S3E by LC/LC-MS/MS**

**Sample preparation**: Samples were supplemented with either 400 μL (GC samples) or 100 μL (SOL samples) of 8 M urea and subjected to 2 rounds of probe sonication. Protein concentrations were determined via Bradford assay and ranged from 7.9 – 20.39 mg/mL. Samples were normalized to 75 μg and spiked with undigested bovine casein at a total of either 15 or 30 pmol as an internal quality control standard. Next, samples were supplemented with 3.2 μL of 20% SDS, reduced with 10 mM dithiolthreitol for 30 min at 32°C, alkylated with 20 mM iodoacetamide for 30 min at room temperature, then supplemented with a final concentration of 1.2% phosphoric acid and 199 μL of S-Trap (Protifi) binding buffer (90% MeOH/100mM triethylammonium bicarbonate (TEAB)). Proteins were trapped on the S-Trap micro cartridge, digested using 120 ng/μL sequencing grade trypsin (Promega) for 1 hr at 47°C, and eluted using 50 mM TEAB, followed by 0.2% FA, and lastly using 50% ACN/0.2% FA. All samples were then lyophilized to dryness.

**TMT Labeling:** Each sample was resuspended in 25 μL 200 mM TEAB pH 8.0. 20 μL were taken of each sample and the remaining was combined to form 3 SPQC pooled samples. Fresh TMTpro 16plex reagents (Thermo, 0.5 mg for each 16plex reagent) was resuspended in 20 μL 100% ACN and 4 μL added to each sample. Samples were incubated for 1 hour at RT. After 1-hour reaction, 1.5 μL of 5% hydroxylamine was added and incubated for 15 minutes at room temperature to quench the reaction. Samples were combined, frozen, and then lyophilized to dryness.

**Fractionation Procedure**. The set of samples were resuspended in 300 μL 0.1% TFA. Samples were fractionated following the PierceTM High pH Reversed-Phase Peptide Fractionation Kit. A total of 8 fractions were collected; combining fractions 1 and 4, fractions 2 and 3 resulted into 6 final fractions. Fractions were frozen and lyophilized overnight. Samples were resuspended in 12 μL 1%TFA/2% ACN prior to LC-MS analysis.

**LC-MS/MS Analysis**. Quantitative LC/MS/MS was performed on 3 μL (25% of sample) using a nanoAcquity UPLC system (Waters Corp) coupled to a Thermo Orbitrap Fusion Lumos high resolution accurate mass tandem mass spectrometer (Thermo) equipped with a FAIMSPro device via a nanoelectrospray ionization source. Briefly, the sample was first trapped on a Symmetry C18 20 mm × 180 μm trapping column (5 μl/min at 99.9/0.1 v/v water/ACN), after which the analytical separation was performed using a 1.8 μm Acquity HSS T3 C18 75 μm × 250 mm column (Waters Corp.) with a 60-min linear gradient of 5 to 30% ACN with 0.1% FA at a flow rate of 400 nanoliters/minute (nL/min) with a column temperature of 55°C. Data collection on the Fusion Lumos mass spectrometer was performed for three difference compensation voltages (-40v, -60v, -80v). Within each CV, a data-dependent acquisition (DDA) mode of acquisition with a r=120,000 (@ m/z 200) full MS scan from m/z 375 – 1600 with a target AGC value of 4e5 ions was performed. MS/MS scans were acquired in the Orbitrap at r=50,000 with a target AGC value of 1e5 and max fill time of 105 ms. The total cycle time for each CV was 1s, with total cycle times of 3 sec between like full MS scans. A 45s dynamic exclusion was employed to increase depth of coverage. The total analysis cycle time for each TMT fraction was approximately 1 hour.

**Quantitative Data Analysis.** Following UPLC-MS/MS analyses, data were imported into Proteome Discoverer 2.5 (Thermo Scientific Inc.) where quantitative signals correlating the TMT16 reporter ions (m/z 126, 127N, 127C, etc.) were extracted. In addition to quantitative signal extraction, the MS/MS data was searched against the SwissProt H. sapiens database (downloaded in Nov 2019), EBNA-LP sequences provided, a common contaminant/spiked protein database (bovine albumin, bovine casein, yeast ADH, etc.), and an equal number of reversed-sequence “decoys” for false discovery rate determination. Mascot Distiller and Mascot Server (v 2.5, Matrix Sciences) were utilized to produce fragment ion spectra and to perform the database searches. Database search parameters included fixed modification on Cys (carbamidomethyl), Lys (TMT), peptide N-termini (TMT) and variable modifications on Met (oxidation), Pro (hydroxyproline), and Ser/Thr/Tyr (phosphorylation). Precusor mass tolerances were 2.0 ppm and product ion mass tolerances were 0.02 da with semi-trypsin enzyme rules required. Peptide Validator and Protein FDR Validator nodes in Proteome Discoverer were used to annotate the data at a maximum 1% protein false discovery rate based on q-value calculations. Note that peptide homology was addressed using razor rules in which a peptide matched to multiple different proteins was exclusively assigned to the protein has more identified peptides. Protein homology was addressed by grouping proteins that had the same set of peptides to account for their identification. A master protein within a group was assigned based on % coverage.
