# Supplementary material for: Epstein-Barr virus protein EBNA-LP engages YY1 through leucine-rich motifs to promote naïve B cell transformation
Source: PLoS Pathog. 2024 Jul 31;20(7):e1011950. doi: 10.1371/journal.ppat.1011950 (PMC11318927; doi:10.1371/journal.ppat.1011950)
Supplement: S1 Table — (DOCX) [file ppat.1011950.s002.docx]

**S1 Table. DNA Sequences of EBNA-LP constructs inserted into trans-complementation vector.**

|  | **DNA Sequence Encoding EBNA-LP** |
| --- | --- |
| EBNA-LP (Wild Type) | ATGGGTGATAGAAGCGAGGGCCCCGGCCCGACAAGACCCGGCCCACCCGGCATCGGGCCCGAGGGACCTCTGGGCCAACTACTCAGACGCCATCGCAGCCCTAGCCCTACGAGAGGCGGCCAAGAGCCGCGGAGAGTGAGGAGAAGAGTGTTGGTGCAGCAAGAGGAAGAAGTCGTGTCGGGGAGCCCAAGCGGCCCAAGGGGTGATAGGTCAGAGGGACCTGGGCCCACCCGTCCTGGGCCGCCCGGTATCGGTCCTGAAGGTCCCCTCGGACAATTACTGAGACGTCATCGTTCTCCTAGCCCAACACGCGGCGGCCAAGAACCTAGGAGGGTGCGACGCCGTGTGCTCGTCCAACAAGAGGAAGAGGTAGTGAGCGGTTCTCCAAGCGGCCCAAGGGGGGATAGATCTGAGGGTCCCGGGCCGACAAGACCCGGGCCCCCCGGTATAGGCCCCGAGGGCCCCCTGGGTCAACTATTACGGCGACATAGAAGCCCCTCGCCCACTAGAGGAGGCCAAGAGCCTAGAAGAGTGAGAAGAAGAGTCCTTGTACAGCAAGAAGAAGAGGTGGTTAGCGGGTCCCCCTCGGGGCCTAGAGGGGATCGATCGGAGGGCCCCGGCCCAACTAGACCGGGTCCGCCCGGAATCGGTCCTGAGGGGCCATTGGGTCAACTGCTGCGAAGACACCGATCACCGTCTCCAACTCGTGGCGGCCAAGAGCCGAGACGAGTGAGGCGAAGGGTTCTCGTGCAACAAGAGGAAGAAGTAGTTAGCGGCAGCCCTAGCGGACCCCTTAGACCTAGACCTAGACCCCCCGCTAGAAGCCTGAGAGAGTGGCTGCTGAGAATCAGAGACCACTTCGAGCCCCCCACCGTGACCACACAGAGACAGAGCGTGTACATCGAAGAAGAAGAAGATGAGGATTGA |
| S3A Mutant | ATGGGGGATAGATCCGAAGGCCCTGGGCCAACACGCCCGGGACCACCCGGAATCGGGCCTGAAGGCCCCCTTGGCCAACTGCTCAGACGCCATAGAGCCCCCGCACCTACGAGAGGCGGCCAAGAGCCGCGGAGAGTGAGGAGAAGAGTGTTAGTTCAGCAAGAGGAAGAGGTAGTTAGTGGGGCCCCGTCCGGGCCGCGTGGGGATAGATCTGAAGGTCCCGGCCCGACGCGTCCCGGCCCCCCGGGGATCGGTCCCGAGGGTCCCTTAGGACAATTACTCCGGAGACATAGAGCCCCCGCCCCAACACGCGGCGGCCAAGAACCTAGGAGGGTGCGACGCCGAGTGCTCGTGCAGCAAGAGGAGGAAGTCGTTAGCGGTGCACCCTCCGGCCCGCGTGGAGATCGCTCTGAAGGGCCCGGACCGACACGGCCCGGCCCCCCCGGCATAGGCCCCGAAGGCCCACTGGGCCAACTATTACGGCGACACAGAGCCCCCGCTCCAACTAGAGGAGGCCAAGAGCCTAGAAGAGTGAGAAGAAGAGTCCTGGTGCAGCAAGAGGAAGAGGTGGTATCGGGGGCTCCCTCGGGGCCTAGAGGCGATCGAAGCGAAGGTCCCGGCCCCACAAGGCCCGGTCCTCCCGGTATAGGGCCTGAAGGGCCCTTAGGCCAACTTTTGCGCCGTCATAGGGCGCCCGCGCCCACAAGAGGCGGCCAAGAGCCGAGACGAGTGAGGCGACGAGTTCTGGTACAACAAGAGGAGGAAGTGGTTAGCGGAGCGCCTAGCGGGCCCCTTAGACCTAGACCTAGACCCCCCGCTAGAAGCCTGAGAGAGTGGCTGCTGAGAATCAGAGACCACTTCGAGCCCCCCACCGTGACCACACAGAGACAGAGCGTGTACATCGAGGAGGAAGAGGACGAGGATTGA |
| S3E Mutant | ATGGGTGATAGATCAGAGGGCCCCGGCCCGACAAGGCCGGGCCCCCCCGGCATCGGCCCCGAGGGCCCCCTGGGCCAACTGCTCAGACGCCATAGAGAGCCGGAGCCTACGAGAGGCGGCCAAGAGCCGCGGAGAGTGAGGAGAAGAGTCTTAGTGCAACAAGAGGAGGAAGTAGTCTCCGGGGAGCCATCCGGCCCTAGAGGGGATCGATCAGAGGGACCCGGCCCTACTAGGCCGGGACCCCCGGGAATAGGTCCGGAAGGTCCCCTCGGACAATTACTCCGGAGACATAGAGAGCCCGAGCCAACACGCGGCGGCCAAGAACCTAGGAGGGTGCGACGCCGTGTACTAGTCCAACAAGAGGAAGAGGTGGTGAGCGGAGAGCCAAGCGGGCCGAGAGGTGACCGGAGCGAGGGGCCCGGCCCGACACGCCCTGGGCCCCCCGGTATAGGCCCCGAGGGCCCACTGGGCCAACTATTACGGCGACATAGAGAGCCCGAGCCCACTCGGGGCGGCCAAGAGCCTAGAAGAGTGAGAAGAAGAGTACTGGTGCAACAAGAGGAAGAGGTGGTATCCGGGGAACCCTCCGGACCAAGGGGGGATCGAAGTGAGGGGCCCGGTCCAACAAGACCTGGTCCACCCGGAATAGGGCCGGAAGGGCCCTTGGGACAGCTGCTACGCAGACATAGAGAACCTGAGCCAACTCGCGGCGGTCAAGAGCCGAGACGAGTGAGGCGAAGGGTTCTAGTTCAACAAGAAGAAGAAGTTGTTAGCGGCGAGCCAAGCGGCCCCCTTAGACCTAGACCTAGACCCCCCGCTAGAAGCCTGAGAGAGTGGCTGCTGAGAATCAGAGACCACTTCGAGCCCCCCACCGTGACCACACAGAGACAGAGCGTGTACATCGAAGAGGAAGAAGACGAAGATTGA |
| LRM Mutant | ATGGGGGACCGGTCGGAAGGCCCCGGCCCGACGAGACCTGGACCCCCCGGGATTGGGCCCGAGGGTCCTGCCGGGCAAGCCGCTAGACGCCATCGTTCCCCTAGCCCTACGAGAGGCGGCCAAGAGCCGCGGAGAGTGAGGAGAAGAGTTTTAGTACAGCAAGAAGAAGAGGTGGTCTCTGGTTCCCCTAGTGGCCCACGTGGGGATCGAAGCGAGGGTCCTGGTCCGACAAGACCCGGCCCACCCGGTATCGGTCCGGAGGGTCCCGCCGGCCAAGCGGCTAGGCGTCATCGTTCTCCTAGCCCAACACGCGGCGGCCAAGAACCTAGGAGGGTGCGACGCCGTGTGCTCGTTCAACAAGAGGAAGAGGTAGTTTCCGGTAGCCCAAGCGGTCCTAGAGGGGATCGGTCTGAAGGTCCCGGGCCAACACGACCGGGGCCCCCGGGAATAGGGCCCGAAGGCCCCGCTGGCCAAGCCGCACGGCGACATAGAAGCCCCTCGCCCACTAGAGGAGGCCAAGAGCCTAGAAGAGTGAGAAGAAGAGTTCTGGTGCAACAAGAAGAAGAGGTGGTTAGCGGATCACCTAGCGGTCCTAGAGGCGATCGTAGTGAGGGACCGGGACCAACTCGCCCCGGTCCCCCCGGCATTGGCCCCGAAGGGCCTGCCGGCCAAGCCGCCCGGCGTCACCGCTCGCCGAGCCCAACAAGAGGCGGCCAAGAGCCGAGACGAGTGAGGCGAAGGGTGCTCGTGCAGCAAGAGGAAGAAGTTGTTAGCGGCAGCCCTAGCGGGCCCTTAAGACCTAGACCTAGACCCCCCGCTAGAAGCGCGAGAGAGTGGGCGGCGAGAATCAGAGACCACTTCGAGCCCCCCACCGTGACCACACAGAGACAGAGCGTGTACATCGAAGAAGAAGAAGATGAGGATTGA |
