# Supplementary figures and images for: Epstein-Barr virus protein EBNA-LP engages YY1 through leucine-rich motifs to promote naïve B cell transformation
Source: PLoS Pathog. 2024 Jul 31;20(7):e1011950. doi: 10.1371/journal.ppat.1011950 (PMC11318927; doi:10.1371/journal.ppat.1011950)

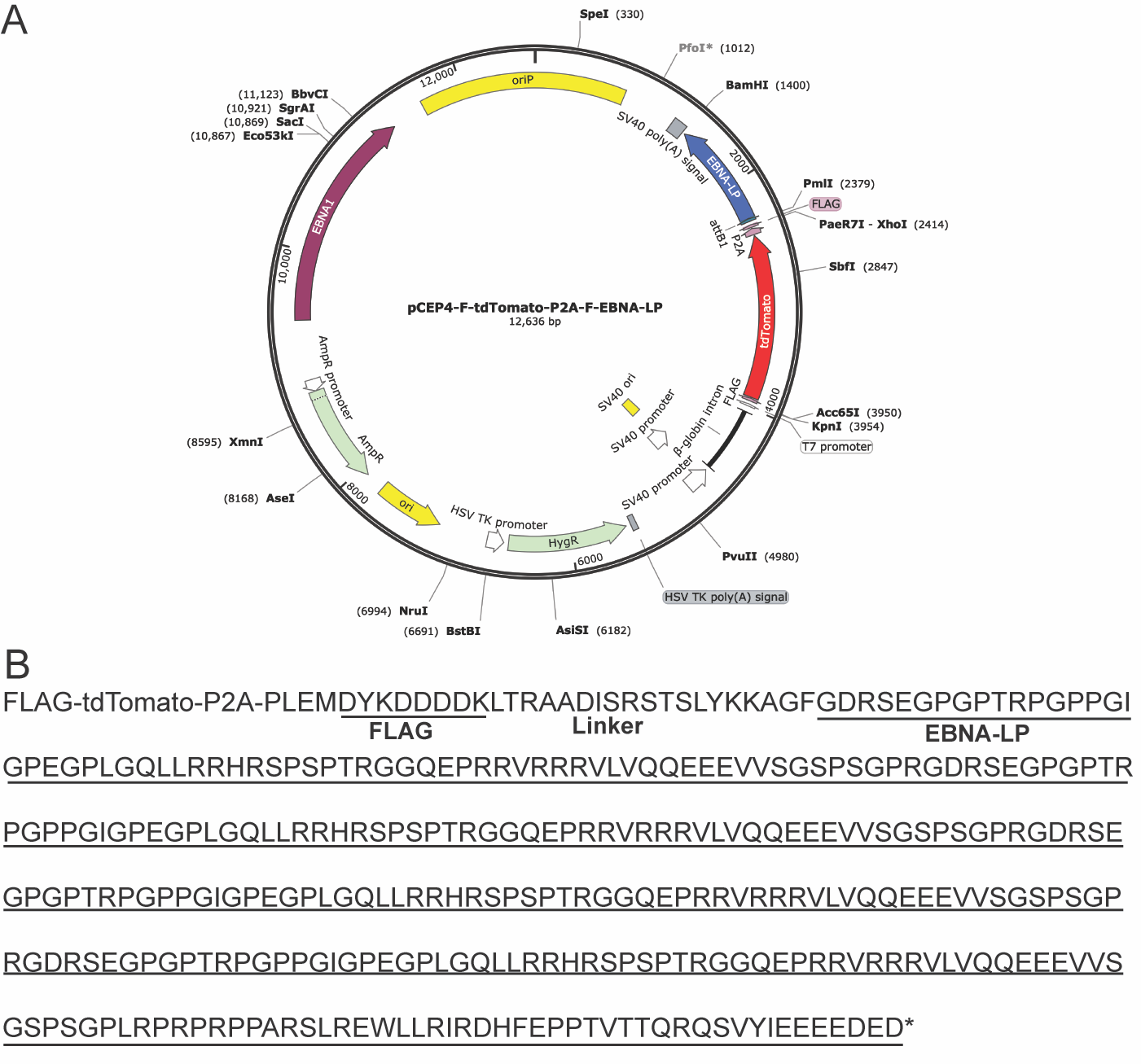

Supplement: S1 Fig — A. Plasmid map of pCEP4 vector encoding FLAG-tdTomato, followed by a P2A cleavage site, and FLAG-EBNA-LP. B. Amino acid sequence of EBNA-LP expressed upon P2A cleavage. FLAG tag and residues encoding EBNA-LP are underlined. (TIF) [file ppat.1011950.s004.tif]

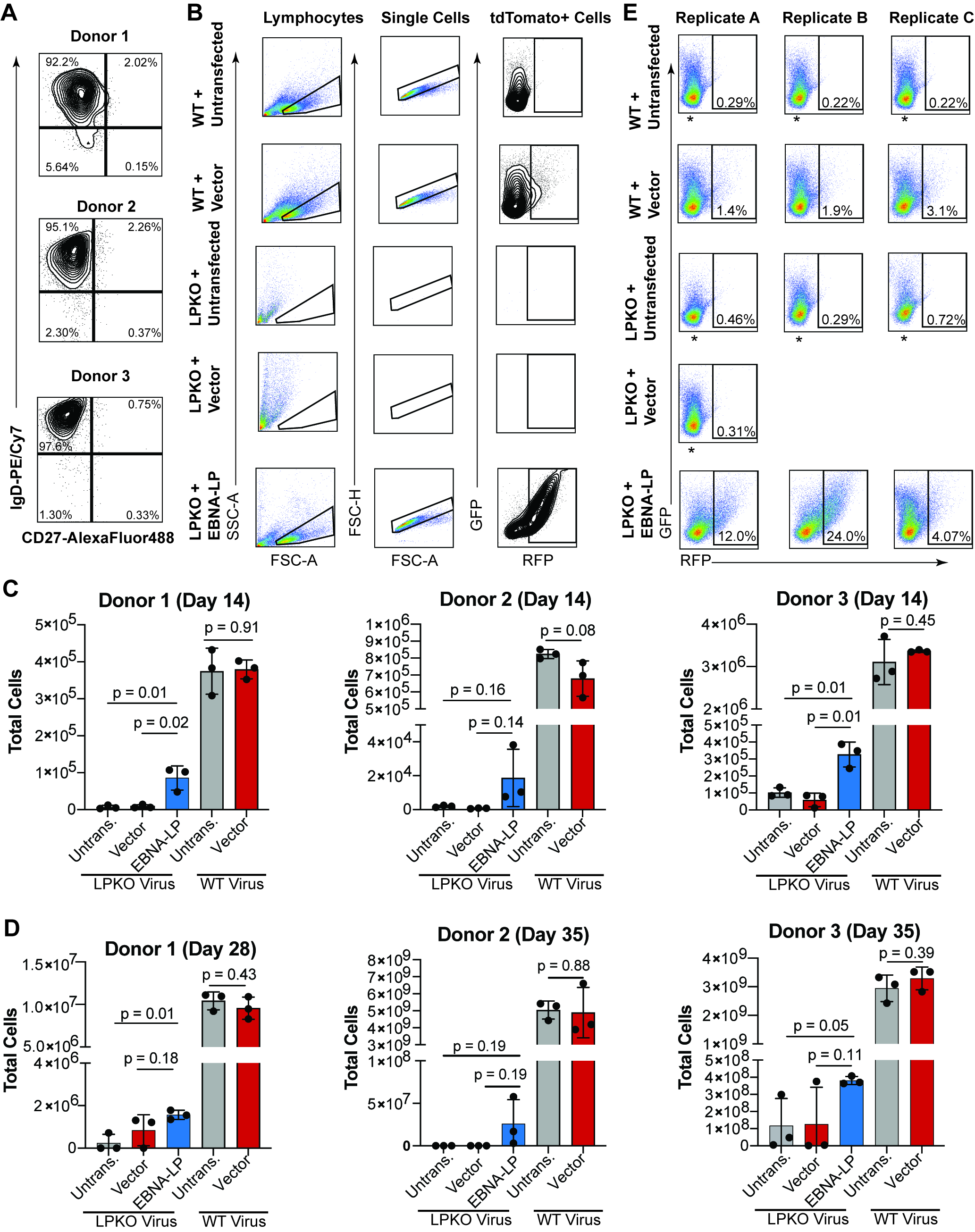

Supplement: S2 Fig — A. Purity of CD19+ B cells in the naïve B cell population from adult blood donors after immunomagnetic selection prior to infection. Naïve B cells are defined as IgD+/CD27-. B. Gating strategy for identifying tdTomato+ cells at each time point. The virus encodes GFP, so this gating strategy is used to remove the majority of false tdTomato+ positives cells from GFP expressing cells. Representative samples from Donor 2 at 35 days post infection are shown. C. Total cells in each condition 14 days post infection for each donor (n = 3). Mean and standard deviation are plotted. P values are determined by unpaired t-test. D. Total cells in each condition at 28 or 35 days post infection (n = 3). E. In Donor 1 and Donor 3, tdTomato negative LCLs grow out in some replicates of LPKO infected untransfected cells, and LPKO infected cells transfected with control vector. Samples from Donor 3 at 35 days post infection are shown. Untransfected conditions were used to define threshold of background for tdTomato negative LCLs. Note some conditions did not lead to outgrowth of LCLs. *Indicates conditions in which cells were transformed into tdTomato negative LCLs. (TIF) [file ppat.1011950.s005.tif]

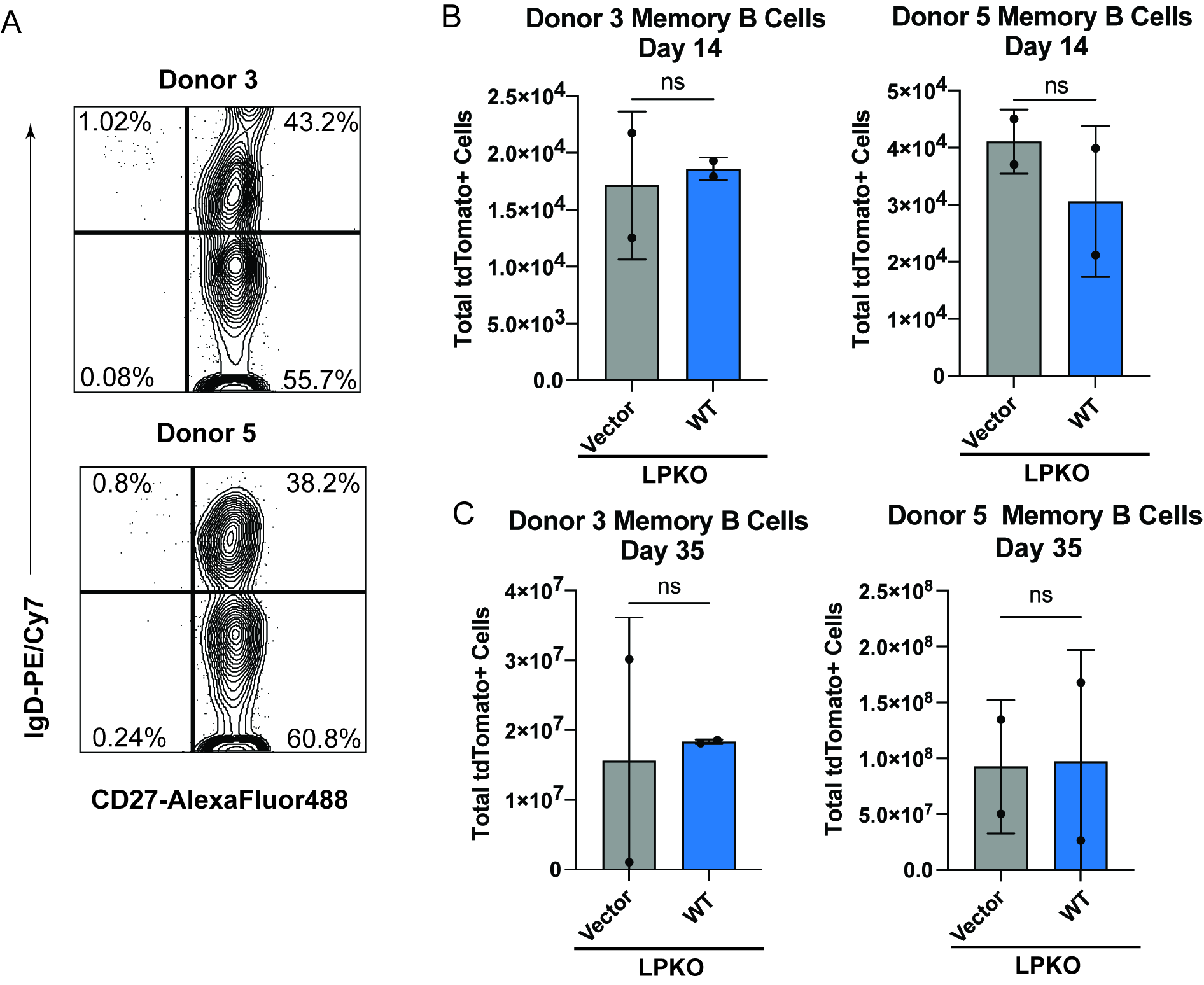

Supplement: S3 Fig — A. Purified of isolated memory B cells. B. Total tdTomato positive cells 14 days post infection. C. Total tdTomato positive cells 35 days post infection. Significance determined by unpaired t-test. (TIF) [file ppat.1011950.s006.tif]

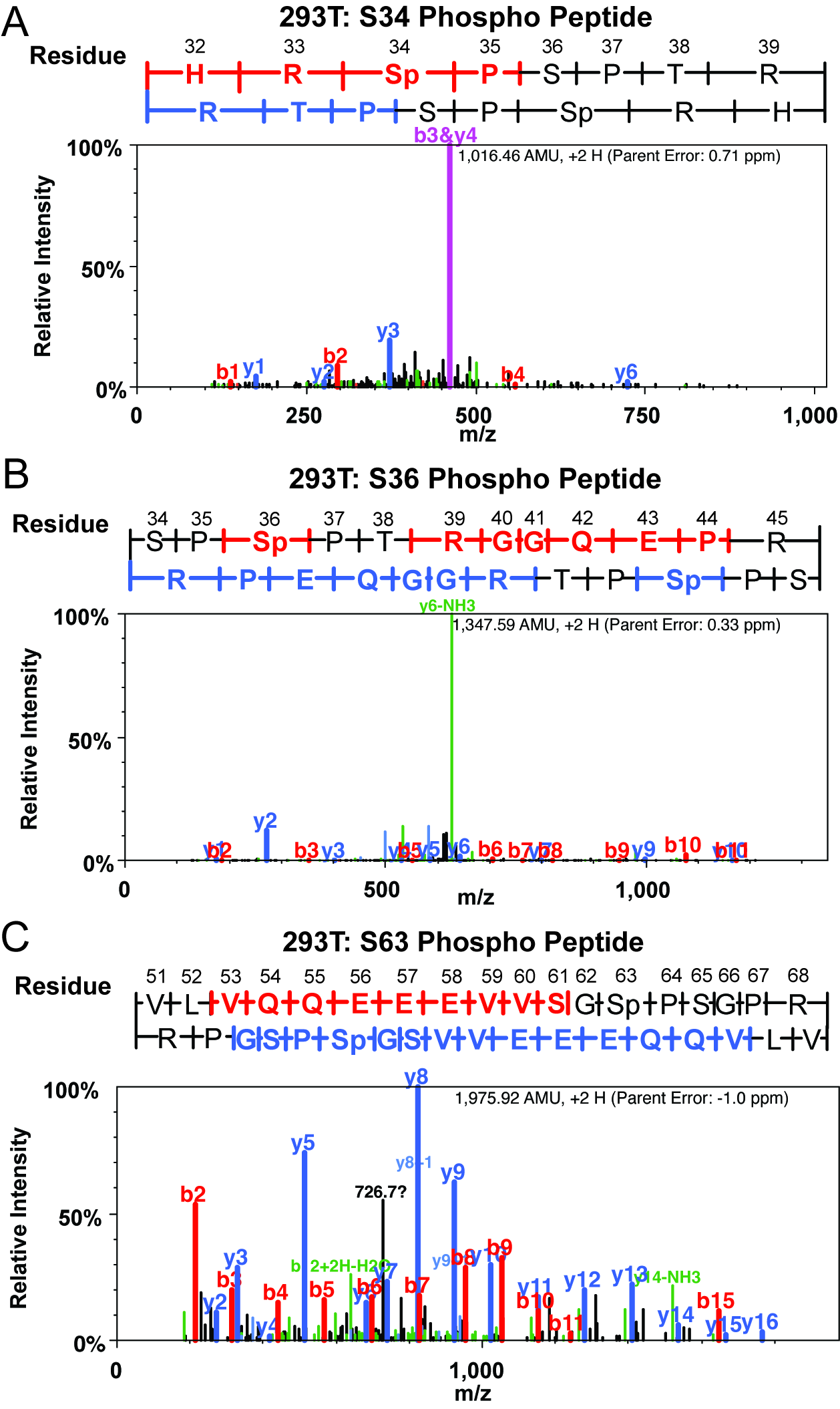

Supplement: S4 Fig — Tandem mass spectra from high-energy collisional dissocation (HCD) fragmentation localizing phosphorylation at sites S34 (A), S36 (B) and S63 (C) collected on a Fusion Lumos Orbitrap mass spectrometer. Localization using site-localizing y/b fragment ions were confirmed within Scaffold PTM using the AScore localization algorithm. (TIF) [file ppat.1011950.s007.tif]

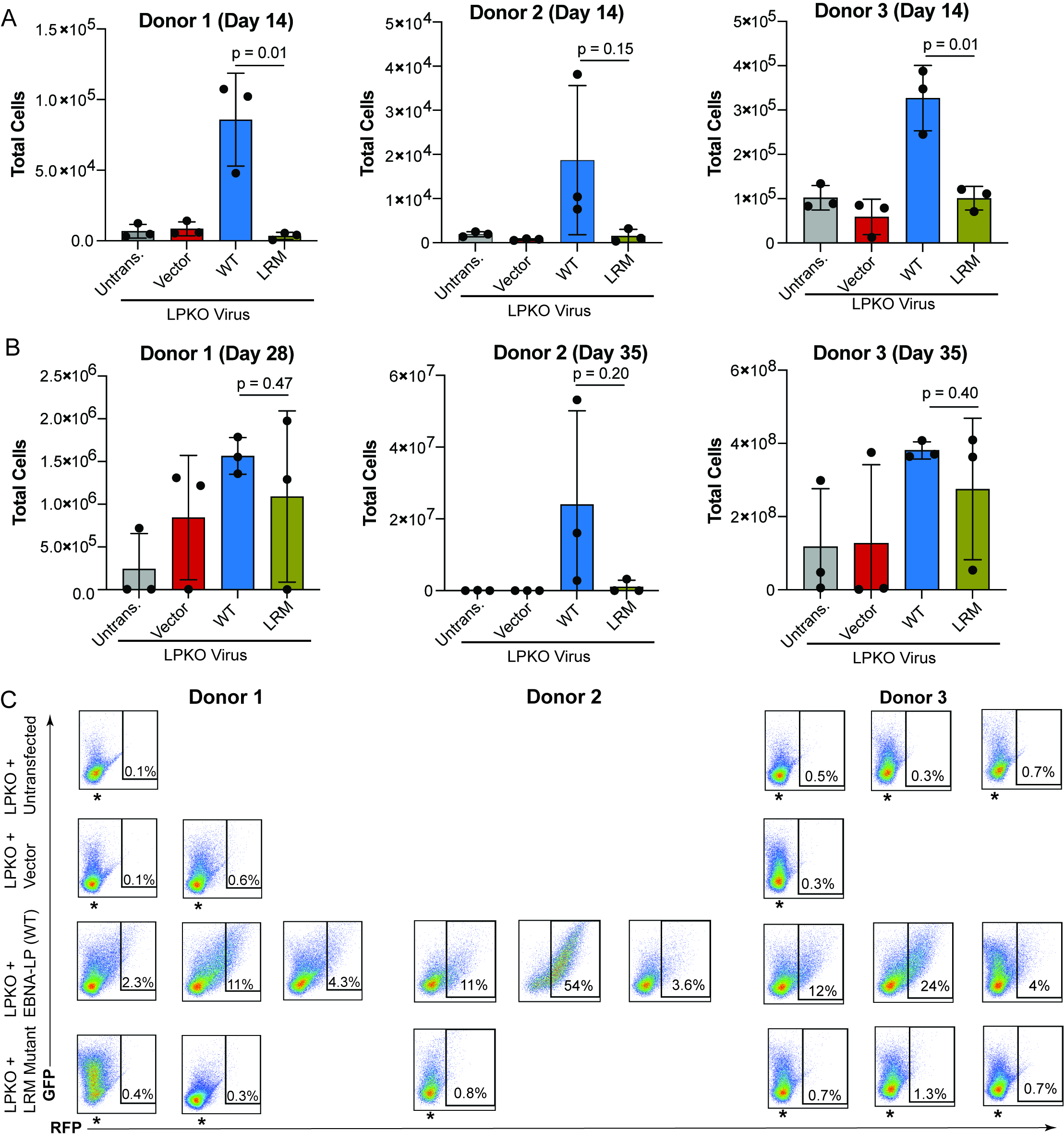

Supplement: S5 Fig — A. Total cells in each condition 14 days post infection for each donor (n = 3). Mean and standard deviation are plotted. P values are determined by unpaired t-test. B. Total cells in each condition at 28 or 35 days post infection (n = 3). C. LCLs at 35 days post infection from adult blood donors. Single cell populations are plotted for tdTomato expression at 4–5 weeks post infection. Percentage of cells that are tdTomato positive as indicated. Note that some conditions did not lead to outgrowth of LCLs. *Indicates tdTomato negative LCLs. (TIF) [file ppat.1011950.s008.tif]

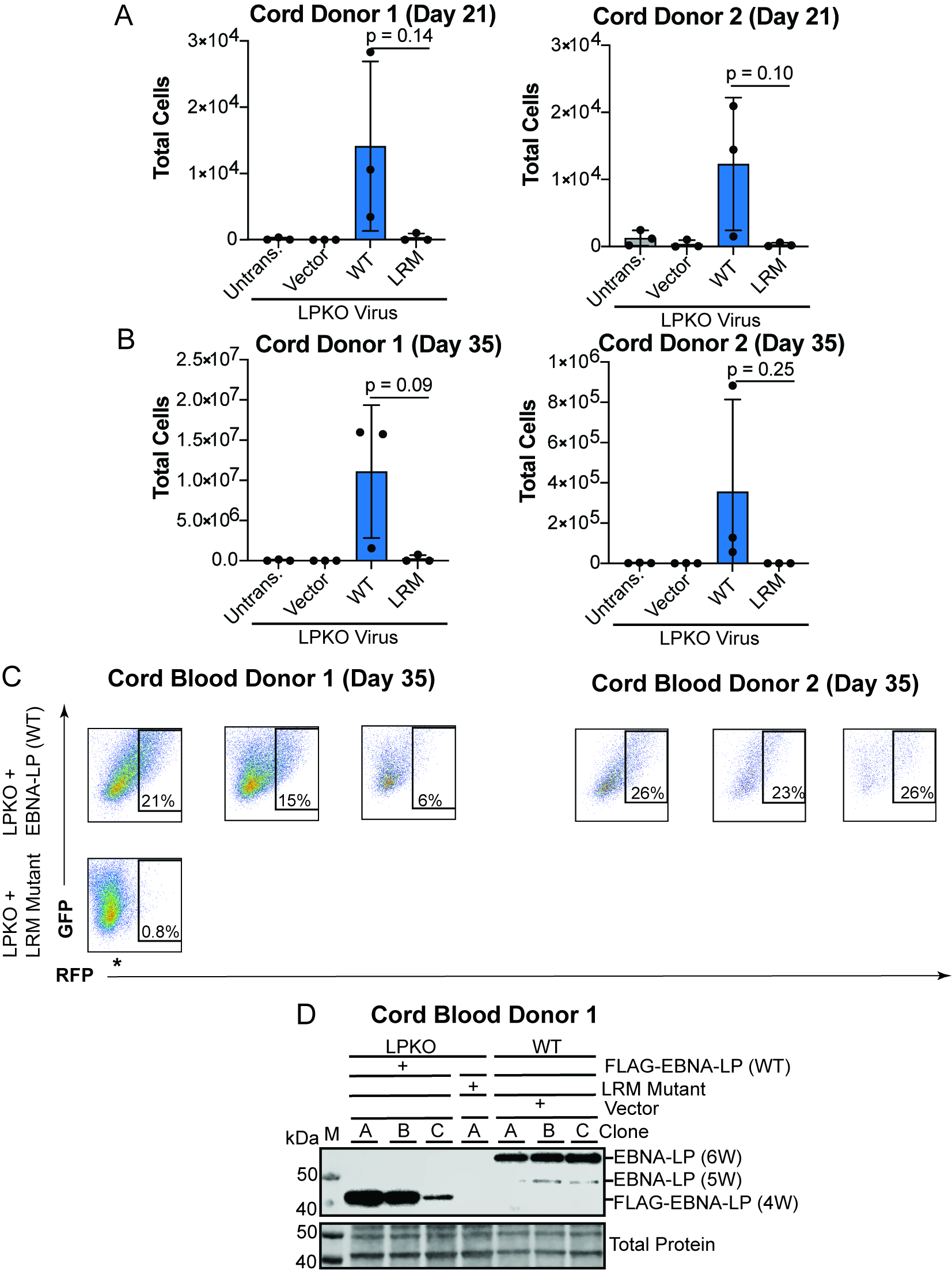

Supplement: S6 Fig — A. Total cells in Cord Blood Donors in each condition 21 days post infection. Mean and standard deviation are plotted. P values determined from unpaired t-test. B. Total cells in Cord Blood Donors 35 days post infection. C. LCLs at 35 days post infection from Cord Blood Donors 1 and 2, at 5 weeks post infection. Percent indicates percent tdTomato positive cells. Note that no cells were transformed into LCLs in either donor from LPKO infected conditions that were either untransfected or transfected with control vector. *Indicates tdTomato negative LCL. D. Western blot of LCLs derived from cord blood donor 1. (TIF) [file ppat.1011950.s009.tif]

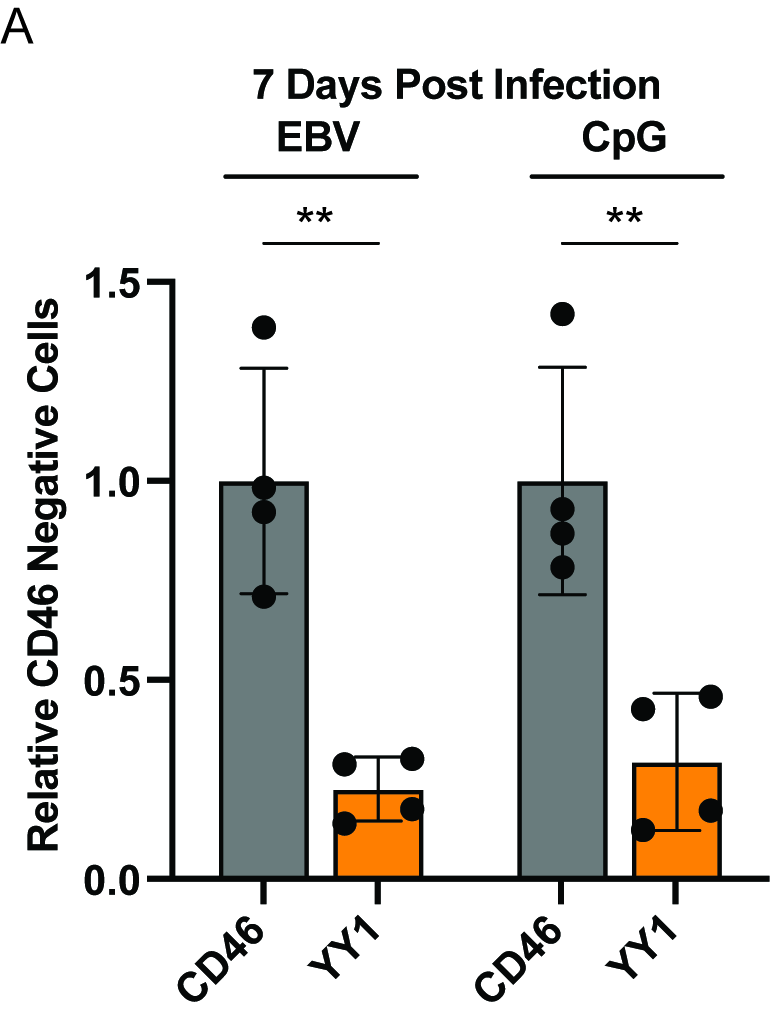

Supplement: S7 Fig — A. Relative number of CD46 negative cells upon EBV infection or CpG stimulation 7 days post infection with CD46 alone or CD46 and YY1 knockout. P-values were calculated using Tukey’s multiple comparisons test. ** Indicates p-values <0.01. (TIF) [file ppat.1011950.s010.tif]

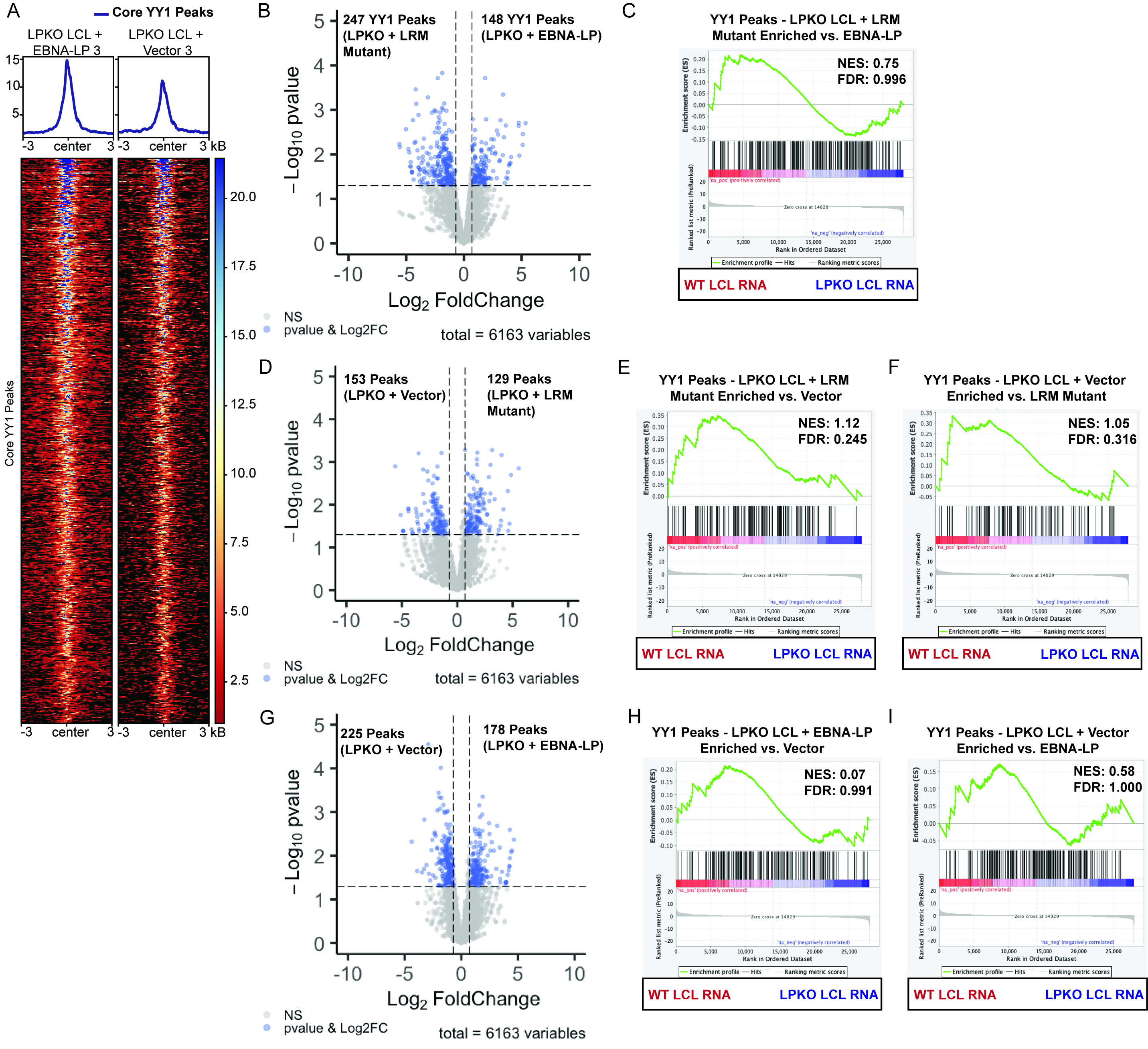

Supplement: S8 Fig — A. Heat map of core the identified 671 core YY1 binding sites in additional replicates for LPKO LCLs trans-complemented with wild type EBNA-LP or control vector. B. Volcano plot of YY1 binding sites enriched in LPKO LCLs trans-complemented with wild type EBNA-LP compared to LRM Mutant. Blue dots indicate significantly enriched sites (Log2 Fold Change above 0.7 and p value above 0.05). C. Enrichment of YY1 peaks enriched in LPKO LCLs trans-complemented with LRM Mutant compared to EBNA-LP in expression data sets from WT and LPKO LCL RNA-sequencing data. D. Volcano plot of YY1 binding sites enriched in LPKO LCLs trans-complemented with LRM mutant compared to vector. E. Enrichment of YY1 peaks enriched in LPKO LCLs LRM Mutant or F. Control vector in WT and LPKO RNA-seq data. G. Volcano plot of LPKO LCLs trans-complemented with EBNA-LP compared to control vector. H. Enrichment of YY1 binding sites enriched in LPKO LCLs trans-complemented with EBNA-LP or I. control vector in WT and LPKO LCLs RNA-seq data. NES = normalized enrichment score. FDR = false discovery rate q-value. Significance is defined as FDR less than 0.05. (TIF) [file ppat.1011950.s011.tif]
