# Supplementary material for: Epstein-Barr virus protein EBNA-LP engages YY1 through leucine-rich motifs to promote naïve B cell transformation
Source: PLoS Pathog. 2024 Jul 31;20(7):e1011950. doi: 10.1371/journal.ppat.1011950 (PMC11318927; doi:10.1371/journal.ppat.1011950)
Supplement: Attachment — Submitted filename: Response to reviewer comments with figures 20240521.docx [file ppat.1011950.s012.docx]

We would like to thank the reviewers for their insightful comments about our manuscript. We have addressed many of the notes critiques and believe that this is now a better study that is ready for resubmission. Our responses to each reviewer are below:

**Reviewer #1:**

1. *The investigators suggest that YY1, perhaps through an interaction with EBNA-LP might play a role in gene regulation or other processes required for immortalization (e.g., regulation of apoptosis), although they state fairly emphatically in parts of the manuscript that this may have nothing to do with EBNA2 transcriptional regulatory functions. However, whether EBNA2 Chip assays overlap with the YY1/EBNA-LP maps is something that still might be worth exploring.*

**Response:** We thank the reviewer for this suggestion, and we appreciate that we had not fully explored the possible role of EBNA2 in the YY1/EBNA-LP genomic association. We have updated figure 6 to include EBNA2 ChIP-Seq data performed in GM12878[1]. We found that while EBNA-LP and YY1 still share many uniquely overlapping peaks, approximately one third of EBNA2 peaks overlap with both EBNA-LP and YY1, and fewer overlap with either YY1 or EBNA-LP alone – highlighting the possibility that together multiple EBNA proteins coordinate YY1 activity during EBV infection. We have updated the results and discussion section to address this (**Fig. 6A-6C**).

Updated Figure 6:

2. *It would be useful to provide the particle to pfu ratio for their virus preparations to get a sense of “gene dosage” that the cells are getting. I can imagine that under biological conditions, naïve B cells may get infected by a single virus particle, where EBNA-LP for example, may be critical—even for EBNA2 transcriptional functions. However, if many of the cells in their current system are receiving 10’s or 100’s (or even 1000’s) of viral genomes, enhancing properties provided by EBNA-LP might be “masked” or even titration of inhibitors of transcription (or immortalization) might be occurring. In addition to gene dosage, “protein dosage” from a number of viral tegument proteins, such as BNRF1, may also negate the requirement of other viral factors during the immortalization process. Commentary is warranted to address this issue as some of their conclusions (role of phosphorylation or even EBNA2 transcriptional enhancement) might require modification if/or when different conditions are used.*

**Response:** Encapsidated viral DNA was extracted and quantified from the virus preparation and BALF5 was used to quantify virus genome numbers. In our LPKO virus preps, infecting at 0.2 RGU is approximately 50 virus genomes per cell where in WT virus preps, 0.2 RGU was approximately 120 virus particles per cell. This RGU was chosen empirically as this lead to nearly all cells being infected. We have updated the methods to include this information and the discussion accordingly to address this caveat. We agree that at high ratios of virus particle to cell, elevated gene dosage and tegument protein may partially mask the requirement for EBNA-LP. We have updated the discussion to address these caveats. However, it should also be noted that in our system, the LPKO fails to immortalize naïve B cells despite a lower number of particles per cell.

3. *The investigators should consider performing a simple experiment to see whether their LRM mutant affects EBNA2 transcriptional coactivation as it still may provide a surrogate assay for investigating EBNA-LP/YY1 mechanistic pathways*.

**Response:** Our trans-complementation assay indicates that EBNA-LP mutants that fail to co-activate EBNA2 are still capable of rescuing LPKO infected naïve B cell outgrowth, while a mutant that does not bind YY1 fails to successfully trans-complement LPKO virus. These results indicate the phenotype of EBNA-LP co-activation is distinct from that of YY1 interaction and co-activation. Therefore, an EBNA2 co-activation assay would not be a surrogate for investigating the mechanism and effects of EBNA-LP interaction with YY1.

4. *Please address why the LRM mutant appears to migrate more slowly in the Immunoblots*.

**Response:** We estimate the observed change in migration between the LRM mutant and wild type EBNA-LP is only equivalent to approximately 1 kDa (**Fig. 5A**). The LRM mutant differs from the wild type EBNA-LP as it contains in total 15 point mutations altering leucines to alanine throughout the protein. These modifications may result in alteration to protein mass enough that when run on protein gels a slower migration observed. We agree this finding is interesting and follow up work will be done to address whether other factors could be contributing to this difference.

**Reviewer #2**

1. *The low consistency is a big problem for this manuscript. In figure 1, 3 and 4, there are 16 bar graphs, only 5 with P value less than 0.05. It is very difficult to draw solid conclusions when the statistics don’t support them. The author should reduce the variation.*

**Response:** We acknowledge that due to the biological variation of primary B cells and transfection efficiency across replicates, the total number of tdTomato positive cells in some donors does not lead to p values below 0.05. However, the most important phenotype we observed is the clear rescue of transformation as observed by tdTomato+ LCL outgrowth from naïve B cell infected with LPKO and trans-complemented. We thank the reviewer for bringing to our attention that we did not provide statistical analysis for this phenotype and have accordingly updated Figures 1-4 to include p-values determined using Fisher’s exact test. From this, we can see trans-complementation with the wild type, S3A, and S3E mutant EBNA-LP is significantly different from vector (**Fig 1E, Fig 2G, Fig 3E, Fig 4C**).

2. *The authors show an important interaction between EBNALP and YY1. However, the authors did not provide any mechanistic insights on why the interaction is important. The authors should at least provide some data on how does this mutant differ from wild-type in regulating gene expression and how do the leucine repeats affect YY1 function*.

**Response:** We thank this reviewer for this suggestion. This manuscript has been updated to include Cut&Run data of YY1 in WT and LPKO LCLs paired with RNA-seq from the same LCLs. From this, we found that a set of YY1 peaks that were unique to WT LCLs and that expression of the set of nearest genes to these peaks were significantly enriched in WT relative to LPKO LCLs, supporting a role for EBNA-LP in modulating YY1 activity as a mechanism of gene transcription activation (**Fig. 6D & 6E**). (See updated figure 6 above.)

We also utilized Cut&Run to examine YY1 binding in LPKO LCLs trans-complemented with wild type EBNA-LP, the LRM mutant, or control vector. We found that reconstituted WT LCLs (LPKO LCLs trans-complemented with wild type EBNA-LP) had overall more YY1 binding sites compared to mutant or vector (**Fig 7D, S8A Fig**), and stronger signal of YY1 binding at peaks shared between all three conditions (**Fig 7E**) – indicating leucine-rich motifs are important for modulating YY1 chromatin-association. We also found that expression of genes nearest YY1 binding sites found in wild type EBNA-LP trans-complemented LPKO LCLs relative to LRM trans-complemented LPKO LCLs were enriched in WT vs LPKO LCLs (**Fig. 7F, S8B-I Fig**), again supporting a role for the leucine-rich motifs in EBNA-LP engaging YY1 and regulating YY1 target gene expression.

New figure 7:

New supplemental figure 8:

3. *The authors should indicate if LCLs are established from transcomplementation.*

**Response:** A table detailing which conditions generate tdTomato positive LCLs, and any conditions that generate tdTomato negative LCLs (likely from non-naïve B cells) are included in Figures 1-4 (Fig 1E, Fig 2G, Fig 3E, Fig 4C).

4. *Figure 1F, 2H, and 5A. The authors should use housekeeping gene Western blot for loading control*

**Response:** REVERT total protein stain is used in place of a house keeping gene as a loading control. Studies have shown total protein stains of various methods are more reliable controls than house keeping genes under many conditions [2-4].

5. *For Figure 1, 3, and 5, the Y axises are rTtomato+cells. Are these the total cell number from an experiment or pool from several wells? For optimum LCL growth, it is better to adjust the concentration to 1x105/ml. The concentration indicated in the figure might not be optimum for LCL growth.*

**Response:** In Figures 1-4 the Y axis corresponds to the total number of tdTomato positive cells per well, not cell density, for each of replicates per experiment. Wells were expanded over time to maintain cell density for each well as appropriate and optimal for outgrowth. We thank this reviewer for bringing to our attention that this may be unclear and have updated the Y axis labels to “Total tdTomato+ Cells” for clarity (Fig 1B-D, Fig 2E & 2F, Fig 3C & 3D, Fig 4A & 4B).

6. *The authors should consider pooling multiple transfections into one datapoint to improve the cell growth or use conditional medium*

**Response:** Transfected cells were initially seeded at approximately 2 million cells per mL after infection with 400,000 cells seeded in 200 uL RPMI with 20% FBS. Cell density was then adjusted over time during outgrowth. We thank the reviewers for this suggestion; however, we do not think the cell growth defect of the LPKO infected cells is due to cell density or growth conditions, but rather due to the importance of EBNA-LP early during infection, particularly in naïve B cells given WT infected cells are effectively transformed under the same conditions. Additionally, we expect transfection efficiency of the trans-complementation constructs is low, approximately 10% across replicates, and thus the low number of tdTomato positive cells reflects that low number of cells successfully trans-complemented.

7. *The authors should include memory B cells in the growth assay to give readers a more precise estimate of the transcomplementation efficiency.*

**Response:** We thank this reviewer for this suggestion. For our B cell subset isolation strategy, we used positive selection for memory B cells and negative selection for naïve B cells (STEMCELL Technologies #17864 ). As a result, we have only been able to consistently obtain 2-3 million memory B cells per donor, which limits the total number of replicates and control samples we have been able to perform for memory B cell trans-complemented. However, using 2 donors (n=2) we found that at both 14 and 35 days post infection the total number of tdTomato positive memory B cells is not significantly different between trans-complementation of LPKO virus with vector or wild type EBNA-LP. This data has been included a new supplemental figure (**S3A-C Fig**). These findings support the ability of memory B cells to be transformed by LPKO virus in the absence of EBNA-LP and highlight the significance of EBNA-LP trans-complementation of LPKO in naïve B cells.

New supplemental figure 3:

**Reviewer #3**

1. *The Cas9 knockout of YY1 is interesting and does show a dependency for B cell growth on YY1, but it is not clear from this experiment that this is an EBV specific dependency, given the broad role of YY1 in apoptosis, nor does it address any EBNA LP specific functional dependency. Could this experiment be done in the LCL complementation background to further investigate dependencies?*

**Response:** We agree that the findings with the YY1 knockout do not show an EBV-specific dependency. We repeated this assay in total B cells and compared the effect of YY1 knockout on the outgrowth of B cells then infected with EBV or stimulated with CpG. 7 days post infection there is a significant and comparable reduction in relative outgrowth of CD46 negative cells when CD46 and YY1 are knocked out compared to CD46 alone under both conditions (**S7 Fig**). Thus, this assay is used to indicate the importance of YY1 broadly in B cells upon activation and suggests the EBV viral proteins mediate YY1 activity because of the important and wide-ranging roles it plays in B cell outgrowth.

New supplemental Figure 7:

2. *If EBNA-LP and YY1 overlap in their binding to chromatin and the interaction with YY1 requires the leucine rich motifs, it would be sensible to examine the binding of EBNA-LP and YY1 to specific promoter sites and show that the leucine rich motif mutant LP no longer binds/shows reduced binding. This would provide further evidence for the interaction and its potential function.*

**Response:** Using YY1 Cut&Run, we found unique YY1 peaks in WT compared to LPKO LCLs that correspond to differentially expressed genes, supporting a role for EBNA-LP/YY1 association in gene expression (**Fig 6D & 6E**). We also investigated YY1 binding in LPKO LCLs trans-complemented with wild type EBNA-LP, LRM mutant, and vector. We found LCLs expressing wild type EBNA-LP had overall more YY1 peaks, stronger YY1 binding at peaks shared by all conditions, and that YY1 peaks enriched in wild type vs. LRM mutant expressing LCLs corresponded to genes most highly expressed in WT LCLs compared to LPKO LCLs (**Fig 7D-F**). Unfortunately, we found EBNA-LP Cut&Run with antibody JF186 to be technically challenging and difficult to reproduce, likely due to the challenges of performing chromatin binding assays such as ChIP-Seq and Cut&Run on a protein that does not directly bind DNA. Future work on this project will be done to optimize parameters in this assay to enable identification of EBNA-LP binding sites on chromatin for wild type and mutant EBNA-LP. (See updated figure 6 and 7, and new supplemental figure 8 above).

3. *Line 222-222 page 10 states that 'these results suggest mutation of the LRMs results in loss of an interaction between EBNA LP and a cellular protein'. I am not sure that this has been adequately demonstrated for this conclusion at this point, just that the mutation impacts on function. Suggest revising this*.

**Response:** We thank reviewers for this suggestion and have revised this sentence accordingly.

1.	Harley JB, Chen X, Pujato M, Miller D, Maddox A, Forney C, et al. Transcription factors operate across disease loci, with EBNA2 implicated in autoimmunity. Nat Genet. 2018;50(5):699-707.

2.	Musyaju S, Modi HR, Flerlage WJ, Scultetus AH, Shear DA, Pandya JD. Revert total protein normalization method offers a reliable loading control for mitochondrial samples following TBI. Anal Biochem. 2023;680:115301.

3.	Kirshner ZZ, Gibbs RB. Use of the REVERT((R)) total protein stain as a loading control demonstrates significant benefits over the use of housekeeping proteins when analyzing brain homogenates by Western blot: An analysis of samples representing different gonadal hormone states. Mol Cell Endocrinol. 2018;473:156-65.

4.	Thacker JS, Yeung DH, Staines WR, Mielke JG. Total protein or high-abundance protein: Which offers the best loading control for Western blotting? Anal Biochem. 2016;496:76-8.
